# Use of the ME-BYO Index, a Mobile Health App, During an Online Strength Training Program in Adults: Fidelity, Feasibility, and Acceptability Study

**DOI:** 10.2196/63123

**Published:** 2025-12-16

**Authors:** Yoshinobu Saito, Naoki Kikuchi, Kaname Watanabe, Sho Nakamura, Hiroto Narimatsu

**Affiliations:** 1 Faculty of Sport Management, Nippon Sport Science University, 1221-1 Kamoshida-cho, Aoba-ku, Yokohama, 227-0033, Japan, 81 45-963-7900; 2 Graduate School of Physical Education, Health and Sport Studies, Nippon Sport Science University, Tokyo, Japan; 3 Faculty of Sport Science, Nippon Sport Science University, Tokyo, Japan; 4 Cancer Prevention and Control Division, Kanagawa Cancer Center Research Institute, Yokohama, Japan; 5 Center for Innovation Policy, Kanagawa University of Human Services, Kawasaki, Japan; 6 Department of Genetic Medicine, Kanagawa Cancer Center, Yokohama, Japan; 7 Graduate School of Health Innovation, Kanagawa University of Human Services, Kawasaki, Japan

**Keywords:** digital health, resistance training, physical fitness, health outcomes, intrinsic capacity, self-measurement, smartphone, surveys and questionnaires, feasibility studies, implementation, Japan

## Abstract

**Background:**

Although various apps have been developed to support health behaviors, they are mostly commercial, possibly limiting the number of users. The ME-BYO index was developed by Kanagawa Prefecture in 2019 to comprehensively and numerically measure and visualize an individual’s current health status and future disease risk by quantifying data. The ME-BYO index is free of charge, so it can be made available to as many prefectural residents as possible for health promotion. Effective online strength training programs are being developed that, when combined with ME-BYO index measurements, will help with both exercise habits and health management.

**Objective:**

In this study, we aimed to validate the fidelity, feasibility, and acceptability of self-measurement using the ME-BYO index during the implementation of an online strength training program.

**Methods:**

Participants were 23 adults aged 40 years or older who did not regularly perform muscle strengthening exercises. The strength training program was performed twice a week for 8 weeks (16 sessions in total), and the ME-BYO index was explained to the participants so that they could self-measure the ME-BYO index with a smartphone on the day of the strength training program, before its implementation. The ME-BYO index during the study period was continuously collected from the app, and the ME-BYO index adherence rate was calculated. Questionnaires were used to assess the feasibility (difficulty of measurement and motivation to improve lifestyle) and acceptability (intention to maintain measurement and appropriate frequency of measurement) of implementing and continuing the ME-BYO index measurements. Changes in the ME-BYO index between the first and second halves of the program period, examination of items strongly related to changes in the ME-BYO index, and a comparison of physical fitness and health outcomes before and after the program period were conducted.

**Results:**

The mean ME-BYO index adherence rate during the strength training program was 89.4% (SD 17%). Regarding acceptability, the participants were highly motivated to continue measuring the ME-BYO index (77%), and the appropriate frequency of measurement was once a week and twice a week (31% and 31%, respectively). Panel data analysis of the self-measured ME-BYO index showed no significant change in the ME-BYO index score; however, it increased to a higher score. Examination of the items that increased the overall score indicated that systolic blood pressure, mental resilience, and Mini-Cog scores were the relevant factors. The pre- and postprogram measurements showed no significant changes in items other than physical fitness.

**Conclusions:**

The fidelity, feasibility, and acceptability of measuring the ME-BYO index during a regular online strength training program were high, suggesting that self-measurement of the ME-BYO index could be used to implement and maintain healthy behaviors. These findings suggest that the ME-BYO index can be recommended as a basic health app.

## Introduction

Appropriate health behaviors are important for maintaining and improving health. In recent years, there have been remarkable developments in mobile health to support healthy behaviors, with various apps being developed [1]. Reviews on the effectiveness of these apps have examined healthy behaviors such as changes in physical activity [2,3], dietary management [4], smoking cessation [5], weight management [6], diabetes management [7], hypertension management [8-10], and medication adherence [11,12], as well as their combined effects with other interventions. However, most of these apps have been commercially developed. Users of smartphone health apps have high health literacy and financial affordability [13,14], and these factors may limit the available population. A review of apps for mental health among underserved young people found that, while acceptance was high among economically disadvantaged residents (with positive evaluations and high satisfaction), they did not intend to continue using the apps. This population was also reluctant to pay for the apps [15].

Furthermore, while commercially available apps have high download numbers and receive positive evaluations regarding ease of use and engagement, significant variations in quality have been reported. Many of these apps are not evidence-based, are inconsistent with public health guidelines, lack empirical evidence of effectiveness or validation of measurement tools, lack theoretical grounding, lack expert and user involvement, and fail to provide assurances around privacy, security, or health risks [16]. Therefore, it is valuable for local governments to develop apps that address these challenges and that all residents can use with confidence.

Kanagawa Prefecture, one of Japan’s largest local governments, defines “ME-BYO” as a state of continuous change between health and disease, rather than a clear distinction between health and disease, and promotes efforts to improve presymptomatic conditions based on three pillars: diet, exercise, and social participation. In March 2020, the prefecture’s health management ME-BYO index, which allows individuals to measure their state of ME-BYO, was released on the smartphone app “My ME-BYO record” [17]. The ME-BYO index is a simple and comprehensive index that can be calculated by entering and measuring 15 items across the areas of metabolic function, locomotor function, cognitive function, and mental resilience. It is expected to promote behavioral changes in individual health and the construction of new systems in society through the concrete quantification of health status [18].

It is unique in that it includes risk factors for dementia (BMI, blood pressure, and mental health) and the Mini-Cog for screening cognitive function. To date, a new longitudinal study based on the Kanagawa Prospective ME-BYO Cohort Study has been initiated to verify the validity and reliability of the measurement method [19,20] and incorporate a future prediction function. However, the real-world feasibility of the ME-BYO index has not yet been validated.

The ME-BYO index was developed for health promotion among residents. This app is provided free of charge to enable widespread use. Therefore, disseminating the app and verifying its feasibility are crucial for addressing health disparities.

Epidemiological studies on physical activity have recently focused on strength training, low-intensity physical activity, and sedentary behaviors. Among these, meta-analyses have shown that strength training reduces the risk of all-cause mortality and development of cardiovascular diseases, cancer, and diabetes [21]. The COVID-19 pandemic has led to the development of various online exercise programs. In particular, online strength training programs, which are convenient and can be completed relatively quickly, are as effective as in-person training and a promising approach for busy working populations [22]. Therefore, combining the measurement of the ME-BYO index with online strength training programs may contribute to motivation to practice exercise and health management.

Therefore, in this study, we aimed to examine the fidelity, feasibility, and acceptability of the ME-BYO index during the implementation of an online strength training program, which is a promising approach for exercise continuity in the working-age population.

## Methods

### Study Design and Participants

This was a prospective single-arm study. The participants were 23 adults aged 40 years or older (41‐68 y) who did not regularly perform muscle strengthening exercises at least twice per week. Information on muscle strengthening exercise habits was collected through self-report.

### Ethical Considerations

This study was approved by the Ethics Committee of the Nippon Sports Science University (023-H043) and by the Ethics Committee of Kanagawa Cancer Center (2023KEN-10). The purpose and content of the study were explained to the participants, and written informed consent was obtained before the study was conducted. Data were collected in a face-to-face setting, and all user data were deidentified before analysis.

### Strength Training Programs

The strength training program was performed twice weekly for 8 weeks (16 sessions in total). Strength training consisted of six exercises (squats, rear raises, dips, lunges, single-leg Romanian deadlifts, and push-ups; Multimedia Appendix 1) and lasted approximately 60 minutes per session. The load was applied with body weight or Therabands, and the number of repetitions per set was 10 to 20, with 1 to 3 sets (low intensity) titrated during the training period. The rest period between sets was 2 minutes. A video call app (ZOOM) was used, and the instructor-to-subject ratio was set at 1 to 5‐7. The strength training program was delivered by well-trained staff [22,23]. The staff members measured the following before and after the strength training program: the ME-BYO index, cognitive function, well-being, psychological distress, physical fitness, body composition, and physical activity.

### Measurement Items

#### The ME-BYO Index

The ME-BYO index was measured by the staff before and after the implementation of the strength training program. During the study period, self-measured ME-BYO index was continuously obtained through the My ME-BYO record app, which was created by Kanagawa Prefecture. The ME-BYO index was measured after a thorough explanation was provided during the preprogram assessments. Instructions for self-measurement were distributed and explained. For this study, data were anonymized, and only information from the study participants was collected.

The ME-BYO index is scored based on a 15-item measure across the following four domains: metabolic function, locomotor function, cognitive function, and mental resilience [18,24]. The World Health Organization categorizes intrinsic capacity into five categories: physical mobility, vitality, psychosocial, sensory, and cognitive [25]. The ME-BYO index was designed using this approach and calculated by summing the weighted partial scores that assessed the status of the four domains. The overall score ranges from 0 to 100 points, with higher scores indicating better health. Various scientific indicators were collected using a survey to construct a scoring formula for domain-specific assessments. The following measurement indices were used to calculate the ME-BYO index: sex, age, BMI, and systolic blood pressure to evaluate metabolic function; the 5-question Geriatric Locomotive Function Scale questionnaire [26] and walking speed measured by a smartphone [19] to assess locomotor function; the 3-question version of the Mini-Cog [20,27,28] to evaluate cognitive function; and individual voice information to evaluate mental resilience. A mind monitoring system (MIMOSYS; PST Inc) was recently developed [29-31] and implemented to evaluate mental resilience using smartphone apps [18].

My ME-BYO record can be installed and used free of charge in the App Store and Google Play. My ME-BYO record and ME-BYO index Terms of Use, Privacy Policy, and Security Policy are available within the app and on the Kanagawa Prefectural Government website [32-34].

#### Fidelity, Feasibility, and Acceptability of the ME-BYO Index Measurement

This study was conducted based on Proctor et al.’s [35] implementation outcome framework (IOF). We examined the individual-level implementation outcomes outlined in the IOF: fidelity, feasibility, and acceptability.

The ME-BYO index self-measurement frequency and adherence rate were calculated as fidelity. The adherence rate was calculated individually as the percentage of the self-measurement to the participation sessions throughout the intervention period, and the average adherence rate was obtained.

In feasibility, they were evaluated using a questionnaire regarding the difficulty of measuring the ME-BYO index twice a week (quite difficult, somewhat difficult, not very difficult, or not at all difficult) and their motivation to improve their lifestyle through regular ME-BYO index measurements (not at all motivated, not very motivated, somewhat motivated, or quite motivated).

The acceptability of the ME-BYO index was evaluated using a questionnaire with the following two items: (1) intention to maintain regular ME-BYO index measurements after the end of the study (not at all, not very much, somewhat agree, or strongly agree); and (2) frequency of ME-BYO index measurement appropriate for the participant (once a year, once every few months, once a month, twice a month, once a week, or at least twice a week). The questionnaire on feasibility and acceptability is detailed in Multimedia Appendix 2.

#### Fidelity and Acceptability of Strength Training Programs

Fidelity was assessed regarding the time required for participation and the adherence rate in the strength training program. Acceptability was evaluated using the following questionnaire on technical difficulty and time allocation of the strength training program: For technical difficulty, participants were asked to choose from the following options: quite difficult, somewhat difficult, not very difficult, or not at all difficult. Time allocation was selected from the following options: not at all appropriate, not very appropriate, somewhat appropriate, and quite appropriate.

#### Cognitive Function

We used the Japanese version of the mild cognitive impairment (MCI) screen, a simple cognitive function scale developed by the Medical Care Corporation, Inc., in the United States, to detect MCI early [36]. The assessment was based on the Memory Performance Index (MPI) score. The MPI score is a unique index that objectively expresses the health status of the brain and is evaluated and calculated using the statistical analysis algorithm of the MCI screen. The higher the MPI score, the higher the patient’s cognitive health status, expressed quantitatively as a number from 0 to 100 [37].

#### Well-Being and Psychological Distress

The World Health Organization-5 Well-Being Index was used to measure the subjective psychological well-being [38]. The respondents answered five statements regarding their feelings over the past 2 weeks. The total score ranged from 0 to 25, with higher scores indicating higher psychological well-being. Scores <13 indicate poor well-being and suggest depression according to the International Statistical Classification criteria [39]. To assess the quality of life, we multiplied the raw score and calculated the percentage ranging from 0 to 100 (low to high quality of life).

The Psychological Distress Scale (K6) score, a robust nonspecific psychological distress measurement tool [40,41], was calculated from six items using a 5-point Likert scale, with the total score ranging from 0 to 24, with higher scores indicating more severe distress. We used the Japanese version of the scale [41], translated and validated from the original scale developed in English [40].

#### Physical Fitness and Body Composition

Grip strength was assessed using a hand grip dynamometer (Takei Scientific Instruments). Participants were allowed two attempts to attain the highest possible rating. They took a standing position and squeezed the dynamometer as hard as possible with one hand, and then repeated the test with the other hand.

A push-up test was performed to evaluate upper extremity muscle function. Participants were required to touch a 10 cm foam pad placed on the floor with their knees on top. The participants were then asked to alternately perform push-ups repeatedly for 30 s, and the number of times they could come to the start position was counted and recorded.

The 5-times chair-standing test required the participants to stand up from a chair without using arms and sit down as quickly as possible. The time required for participants to perform this action was measured 5 times using a stopwatch [42].

Squat and countermovement jumps were measured using a MULTI JUMP TESTER instrument (DKH Co., Ltd). All the participants were instructed to jump as high as possible and were not allowed to use their arms. The highest score of the two trials was accepted as the jump score (cm).

The sit-and-reach flexibility test is widely used as a general test of trunk flexibility and is performed using a digital measuring device (Takei Scientific Instruments). The participants were instructed to sit on the floor and stretch their torso and arms out in front of them with their knees straight. The participants were allowed 2 to 3 attempts to attain the highest possible rating.

Body composition was measured in terms of muscle thickness and body fat content. The muscle thickness (cm) of the triceps brachii (ie, 60% distal between the lateral epicondyle of the humerus and the acromial process of the scapula) was measured using B-mode ultrasound (FAMUBO-D; SEIKOSHA). Body fat (%) was measured using a body composition measuring device (Inbody270; InBody Japan Inc). Height (seca 213; seca Nihon) and weight (HD-665; TANITA corporation) were measured, and BMI (kg/m^2^) was calculated. Systolic blood pressure and diastolic blood pressure (mmHg) were measured using a sphygmomanometer (HEM-7120; OMRON Healthcare Co. Ltd). Participants rested for at least 5 minutes in a seated position before the measurement was taken. They took two readings themselves. If there were no issues, the second reading was used.

#### Physical Activity

Physical activity was measured using a validated accelerometer (Active Style Pro HJA-750C; OMRON Healthcare) [43-45]. Participants were instructed to wear the accelerometer on their waist for 7 consecutive days from when they walked up to when they went to bed, except when bathing, swimming, or other water exposure. The time the accelerometer was worn was calculated by subtracting the time the accelerometer was not worn from 24 h per day. Those with at least 4 days of at least 10 hours of wear per day were included. The outcome measures were average moderate-to-vigorous physical activity time (minutes per day, ≥3.0 METs) and average sedentary time (minutes per day, ≤1.5 METs). A macro program (version 190829), developed and distributed by the Japan Physical Activity Research Platform [46], was used to process the accelerometer data.

### Statistical Analysis

For the panel data analysis of the ME-BYO index self-measured, the means of the first 4 weeks of the program and the second 4 weeks of the program were compared for the overall score, metabolic function score, locomotor function score, cognitive function score, and mental resilience score using an appropriate *t* test. A multilevel analysis was also performed, with the ME-BYO index overall score as the dependent variable; BMI, systolic blood pressure, mental resilience score, Mini-Cog score, 5-question geriatric locomotive function scale, and walking speed as the independent variables; and sex and age as the adjusted variables. Paired *t* tests were used to compare the means of each item in the pre- and postprogram measurements. Statistical analyses were performed using SPSS version 27 software (IBM Corp). The significance level was set at 5%.

## Results

Of the 23 participants, 19 participated in the premeasurement period. Therefore, the 19 participants for whom pre- and postassessments were available were included in the analysis. The baseline characteristics of the study participants are shown in Table 1. The mean age was 57.6 (SD 7.8) years.

The results of the fidelity, feasibility, and acceptability of the ME-BYO index measurement and strength training program are shown in Table 2. Regarding the number of self-measurements and the adherence rate of the ME-BYO index, 13 participants were included in the evaluation because 8 participants had not yet taken the measurement owing to inadequate smartphones or measurement environments. The mean number of measurements was 14.1 (SD 3.1), and the ME-BYO index adherence rate was 89.4% (17%). Regarding self-measurement of the ME-BYO index twice a week during the study period, a few participants had difficulty measuring it (n=5, 39%), and many participants were motivated to improve their lifestyle (n=8, 61%). The intention to maintain the ME-BYO index twice a week was high (n=10, 77%), and the most common appropriate frequency for measuring the ME-BYO index was once or twice a week (n=8, 62%). The adherence rate in the strength training program was 94% (8.5%). The technical difficulty was somewhat difficult (n=9, 47%) but not very difficult (n=7, 37%). Time allocation was appropriate (n=16, 84%).

**Table 1.** Characteristics of the study participants (n=19).

Characteristics	Value, n (%)
Age (y)	
41‐49	3 (15.8)
50‐59	7 (36.8)
60‐68	9 (47.4)
Sex	
Male	8 (42.1)
Female	11 (57.9)
Education (y)	
12‐14	3 (15.8)
16	8 (42.1)
18+	8 (42.1)

**Table 2.** Fidelity, feasibility, and acceptability of the self-measured ME-BYO index and the strength training program.

Item	Value
Fidelity	
Self-measurement of ME-BYO index, mean (SD)	
Number of measurements (times)	14.1 (3.1)
Adherence rate (%)	89.4 (17.0)
Strength training program, mean (SD)	
Participation (times)	15.1 (1.4)
Adherence rate (%)	94.0 (8.5)
Feasibility	
Difficulty of implementing twice-weekly ME-BYO index measurements, n (%)	
Quite difficult	1 (8)
Somewhat difficult	4 (31)
Not very difficult	2 (15)
Not at all difficult	6 (46)
Motivation to improve lifestyle by ME-BYO index measurement, n (%)	
Not at all motivated	1 (8)
Not very motivated	4 (31)
Somewhat motivated	5 (38)
Quite motivated	3 (23)
Acceptability	
Intention to maintain ME-BYO index measurement, n (%)	
Not at all	2 (15)
Not very much	1 (8)
Somewhat agree	8 (62)
Strongly agree	2 (15)
Appropriate frequency of ME-BYO index measurement, n (%)	
Once a year	0
Once every few months	2 (15)
Once a month	1 (8)
Twice a month	2 (15)
Once a week	4 (31)
At least twice a week	4 (31)
Technical difficulty of the strength training program, n (%)	
Quite difficult	0
Somewhat difficult	9 (47)
Not very difficult	7 (37)
Not at all difficult	3 (16)
Time allocation fo ther strength training program, n (%)	
Not at all appropriate	0
Not very appropriate	0
Somewhat appropriate	3 (16)
Quite appropriate	16 (84)

When comparing the mean scores of the ME-BYO index self-measurement between the first and second 4 weeks, there was no significant difference, but there was a change to a higher value (Table 3).

A multilevel analysis using panel data in the self-measured ME-BYO index to examine the measures that increased the overall score revealed that systolic blood pressure, mental resilience, and the Mini-Cog score were significantly associated factors. An increase of 1 mmHg in systolic blood pressure tended to decrease the overall score by 0.15 points. A 1-point increase in mental resilience tended to increase the overall score by 0.10 points. A 1-point increase in Mini-Cog score tended to increase the overall score by 3.57 points (Table 4).

Significant improvements in physical fitness were observed in the postprogram measurements compared with the preprogram measurements, including push-ups, 5-time chair standing test, squat jump, and countermovement jump. No significant changes were observed in the other items (Table 5).

**Table 3.** Changes in the ME-BYO index self-measurement scores^a^.

Item	First half of the program, mean (SD)	Second half of the program, mean (SD)	*P* value
Overall score (points)	91.6 (4.2)	92.9 (4.1)	.15
Metabolic function score (points)	90.2 (8.1)	90.2 (8.2)	.99
Locomotor function score (points)	97.3 (7.9)	97.2 (6.6)	.91
Cognitive function score (points)	96.5 (8.0)	98.7 (2.2)	.22
Mental resilience score (points)	70.3 (19.7)	78.2 (11.2)	.08

a Paired *t*-test: panel data on ME-BYO index self-measurement to compare mean scores for the first 4 weeks and second 4 weeks of the program.

**Table 4.** Measures associated with an increase in the overall score of the ME-BYO index^a^.

	Estimated value	SE	*P* value	95% CI	
BMI (kg/m^2^)	−0.25	0.31	.43	−0.95 to 0.44	
Systolic blood pressure (mmHg)	−0.15	0.02	<.001	−0.19 to −0.12	
Mental resilience score (points)	0.10	0.01	<.001	0.08 to 0.12	
Mini-Cog score (points)	3.57	0.71	<.001	2.15 to 5.00	
Five-question geriatric locomotive function scale (points)	−1.54	1.18	.20	−3.91 to 0.83	
Walking speed (m/s)	−0.01	0.07	.87	−0.16 to 0.14	
Sex: women (reference: men)	−1.68	3.23	.62	−9.24 to 5.88	
Age (years)	−0.04	0.21	.85	−0.54 to 0.46	
Intercept	95.45	12.60	<.001	66.99 to 123.91	

a Multilevel analysis: Using panel data in the self-measurement of the ME-BYO index, the overall score was the dependent variable; BMI, systolic blood pressure, mental resilience score, Mini-Cog score, 5-question geriatric locomotive function scale, and walking speed were the independent variables; and sex and age were the adjusted variables.

**Table 5.** Changes in each evaluation item before and after the online strength training program^c^.

	Before, mean (SD)		After, mean (SD)		*P* value
ME-BYO index (score)	85.4 (7.7)		84.3 (6.2)		.54
BMI (kg/m^2^)	23.6 (4.3)		23.5 (4.2)		.44
Systolic blood pressure (mmHg)	124.4 (21.9)		124.4 (17.9)		.99
Diastolic blood pressure (mmHg)	77.5 (10.5)		81.0 (11.1)		.20
MPI^a^ (points)	69.1 (8.2)		69.6 (8.1)		.65
WHO-5^d^ (points)	18.9 (3.9)		18.7 (3.9)		.81
K6 (points)	7.2 (1.9)		7.6 (2.3)		.19
Grip strength					
Right (kg)	32.3 (10.4)		31.6 (10.4)		.05
Left (kg)	30.3 (11.1)		30.6 (10.9)		.69
Push-up (times/30 s)	15.2 (6.4)		19.2 (6.1)		<.001
5 times chair standing test (sec)	6.4 (1.4)		5.7 (0.6)		.01
Squat jump (cm)	19.8 (5.3)		21.1 (5.6)		.02
Countermovement jump (cm)	22.8 (5.9)		23.9 (6.1)		.045
Sit-and-reach test (cm)	38.1 (7.9)		38.1 (8.6)		.94
Muscle thickness (cm)	3.3 (0.5)		3.3 (0.9)		.77
Body fat (%)	27.1 (6.6)		26.0 (7.7)		.07
MVPA^b^ (min/d)	83.0 (41)		75.7 (39)		.29
Sedentary time (min/d)	542.3 (132.1)		526.3 (125.1)		.33

a MPI: Memory Performance Index.

b MVPA: moderate-to-vigorous-intensity physical activity.

c Paired *t*-test.

d WHO-5: The World Health Organization-5 Well-Being Index.

## Discussion

### Principal Findings

In this study, we evaluated the fidelity, feasibility, and acceptability of self-measuring the ME-BYO Index during an online strength training program. The mean number of measurements was 14.1 (SD 3.1), and the adherence rate was 89.4% (17%), suggesting high fidelity in an 8-week (16 sessions), twice-weekly training program. Although 5 (39%) participants had difficulty measuring the ME-BYO index, 8 (61%) participants were motivated to improve their lifestyle. The importance of daily physical condition and health management was described in the individual questionnaire (Multimedia Appendix 3), suggesting that these factors led to a high number of ME-BYO index measurements. A study on the fidelity of a smartphone nutrition app (MyNutriCart) for making smart and healthy choices when purchasing food in grocery stores found that the average use of the app was 75% for each purchase, with a 69% likelihood of use [47]. This was an 8-week intervention in which participants were instructed to use the app every time they went grocery shopping or at least once a week [48]. Reviews of self-monitoring mental health apps also report adherence rates of 85% to 100%, suggesting high engagement with these apps [49]. Despite differences in input items and duration, this study demonstrated fidelity similar to that of other studies.

Eight participants were unable to self-measure, not due to a lack of interest or motivation but primarily because their smartphones were incompatible. Furthermore, all their baseline characteristics were comparable (Multimedia Appendix 4). Therefore, it is unlikely that this resulted in a biased sample. However, it should be noted that the ME-BYO index measurement was requested on the day of program participation, which may have motivated participants to continue the measurement. Additionally, smartphone literacy may have influenced the results. Concerns remain, particularly among older adults, that limited familiarity with technology may affect outcomes. This highlights the need for further research.

High fidelity was also confirmed for the strength training program (94%), suggesting that the program may be an effective gateway to exercise habits in the working population. Pre- and postprogram measurements revealed an increase in physical fitness. However, no changes were observed in the mental or psychological factors assessed besides the ME-BYO index. These results suggest that, although the evaluation of the ME-BYO index measures not linked to the program is problematic, combining the program with a regular and highly feasible program is effective for regular ME-BYO index measurements. The intention to continue measuring the ME-BYO index twice a week was high (n=10, 77%), and the appropriate frequency of the ME-BYO index measurement was once or twice a week (n=8, 62%), suggesting that continuous ME-BYO index measurement at short intervals could be used as an indicator of the effectiveness of regular exercise and other programs. A prior study using real-world data from the My ME-BYO Record app reported low engagement among repeat users [50]. In contrast, the present study observed high adherence rates when the app was integrated within a structured online strength training program. These findings suggest that combining the app with guided health interventions may be an effective strategy to enhance user engagement and address the low adherence typically observed in free-living settings compared to structured programs.

When comparing the mean ME-BYO index self-measured scores between the first and second 4 weeks, there was a general change to higher scores, although there were no significant differences. Notably, the mental resilience score showed an upward trend, while the scores of the other indicators remained high. The multilevel analysis showed that the measures that increased overall scores were systolic blood pressure, mental resilience, and Mini-Cog scores. The ME-BYO index app has an immediate feedback and advice function for measurement results, and the ability to self-monitor is thought to be a factor that increases its fidelity, feasibility, and acceptability. Self-monitoring is one of the most commonly used behavioral change techniques in health apps [51]. A meta-analysis by Michie et al [52] on behavior change techniques (BCTs) that contributed to improved physical activity and dietary habits found that the most effective BCT among 122 intervention studies was immediate self-monitoring of behavior, indicating the importance of implementing BCTs. This study demonstrated the importance of implementing BCTs.

In a study evaluating the quality of physical activity apps, only 1 out of 65 randomly selected apps that met the criteria had a noncommercial affiliation (government agency). Privacy and security assessments revealed that 29.2% of the apps had no privacy policy. Most apps collect personally identifiable information and share user data with third parties, and more than half do not specify how to ensure data security [53]. Furthermore, only 12 of the 65 apps had a peer-reviewed study associated with them, and only one app was evaluated for efficacy in a trial. This suggests that the quality of commercially developed apps is inadequate. Importantly, this study confirmed the feasibility of a reliable app provided free of charge by the local government, which can be recommended for use as a basic health app.

### Limitations

This study had several limitations. First, it used a before-and-after comparative design, and the influence of psychological bias (the Hawthorne effect) and selection bias cannot be excluded. Second, the sample size was small, which may have resulted in insufficient statistical power. Future studies involving control groups are required. Third, although the evaluation was based on the IOF, the feasibility and acceptability indicators were not validated scales, which may have introduced interpretation bias. Fourth, the study was conducted over a short period. The ME-BYO index includes indices (cognitive and locomotor functions) that do not change easily over a short period. Therefore, it may be difficult to detect the effects of short-term measurements at short intervals, and long-term validation needs to be addressed. The impact on long-term fidelity should also be considered.

### Conclusion

We investigated the fidelity, feasibility, and acceptability of self-measurement of the ME-BYO index during the implementation of an online strength training program in adults with no exercise habits. The fidelity, feasibility, and acceptability of measuring the ME-BYO index during implementation were high, suggesting that self-measurement of the ME-BYO index could be used as a tool for implementing and maintaining healthy behaviors. The fact that these findings were confirmed in an app provided free of charge by the public sector is significant and suggests that it can be recommended as a basic health app.

## Supplementary material

10.2196/63123 Multimedia Appendix 1 Picture of the strength training program.

10.2196/63123 Multimedia Appendix 2 Questionnaire of feasibility and acceptability.

10.2196/63123 Multimedia Appendix 3 Reasons for intention to maintain the ME-BYO index measurement.

10.2196/63123 Multimedia Appendix 4 Comparison of baseline characteristics between the presence and absence of self-measurement.

## Abbreviations

IOF: implementation outcome framework
MCI: mild cognitive impairment
MPI: Memory Performance Index
WHO-5: World Health Organization-5 Well-Being Index
