# Supplementary material for: Use of the ME-BYO Index, a Mobile Health App, During an Online Strength Training Program in Adults: Fidelity, Feasibility, and Acceptability Study
Source: JMIR Hum Factors. 2025 Dec 16;12:e63123. doi: 10.2196/63123 (PMC12707806; doi:10.2196/63123)

Multimedia Appendix 1: Picture of the strength training program

A) Squat

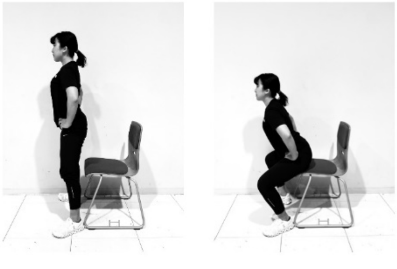

B) Lunge

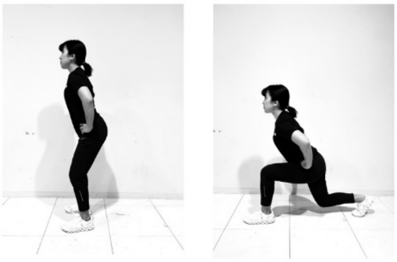

C) Romanian Dead Lift

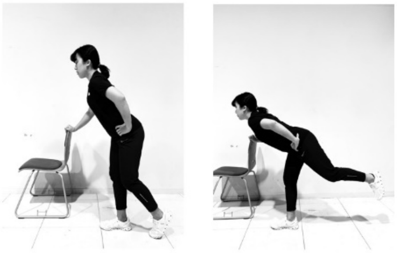

D) Rear Raise

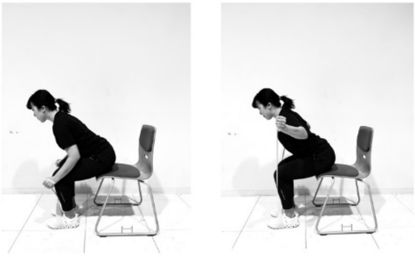

E) Dips

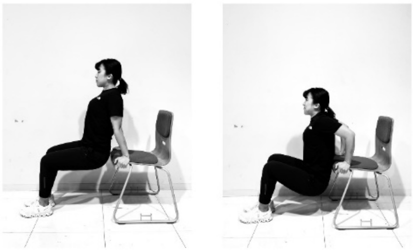

F) Push UP

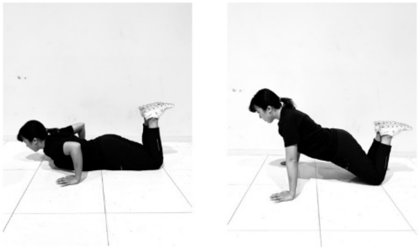

Supplement: Multimedia Appendix 1 [file humanfactors-v12-e63123-s001.pdf]
