# Supplementary material for: Use of the ME-BYO Index, a Mobile Health App, During an Online Strength Training Program in Adults: Fidelity, Feasibility, and Acceptability Study
Source: JMIR Hum Factors. 2025 Dec 16;12:e63123. doi: 10.2196/63123 (PMC12707806; doi:10.2196/63123)
Supplement: Multimedia Appendix 2 [file humanfactors-v12-e63123-s002.pdf]

Multimedia Appendix 2: Questionnaire of Feasibility and Acceptability

Feasibility

Difficulty of implementing twice-weekly ME-BYO index measurements

Q: How difficult was it to regularly measure the ME-BYO index (twice a week)?

A: Quite difficult, Somewhat difficult. Not very difficult, Not at all difficult

Motivation to improve lifestyle by ME-BYO index measurement

Q: Did regularly measuring the ME-BYO index motivate you to improve your lifestyle habits?

A: Not at all motivated, Not very motivated, Somewhat motivated, Quite motivated

Acceptability

Intention to maintain ME-BYO index measurement

Q: After this study ends, do you intend to continue measuring the ME-BYO index twice a week?

A: Not at all, Not very much, Somewhat agree, Strongly agree

Appropriate frequency of ME-BYO index measurement

Q: How often do you think it is appropriate to measure the ME-BYO index?

A: Once a year, Once every few months, Once a month, Twice a month, Once a week, At least twice a week

Technical difficulty of the strength training program

Q: Was the strength training program technically difficult?

A: Quite difficult, Somewhat difficult, Not very difficult, Not at all difficult

Time allocation for strength training program

Q: Was the time allocation for the strength training program appropriate?

A: Not at all appropriate. Not very appropriate, Somewhat appropriate, Quite appropriate
