# Supplementary material for: Use of the ME-BYO Index, a Mobile Health App, During an Online Strength Training Program in Adults: Fidelity, Feasibility, and Acceptability Study
Source: JMIR Hum Factors. 2025 Dec 16;12:e63123. doi: 10.2196/63123 (PMC12707806; doi:10.2196/63123)
Supplement: Multimedia Appendix 3 [file humanfactors-v12-e63123-s003.pdf]

Multimedia Appendix 3: Reasons for intention to maintain the ME-BYO index measurement

Answers for intention to maintain	Reason (free text: original posted)
Not at all	Because it takes time.
Not at all	Daily, I feel that just measuring blood pressure is sufficient.
Not very much	No real sense of score.
Somewhat agree	To know the condition of the body
Somewhat agree	Effects unknown
Somewhat agree	I don't know how effective it is.
Somewhat agree	As a consistent part of physical health care
Somewhat agree	I was aware of the results because I could compare them with the previous results.
Somewhat agree	For healthy life expectancy
Somewhat agree	Manage your own physical condition.
Somewhat agree	I have some idea of my health status.
Strongly agree	If it's a record of your health, it's a record of your health.
Strongly agree	I want to develop an exercise habit to slow down the aging process.
