# Supplementary material for: Use of the ME-BYO Index, a Mobile Health App, During an Online Strength Training Program in Adults: Fidelity, Feasibility, and Acceptability Study
Source: JMIR Hum Factors. 2025 Dec 16;12:e63123. doi: 10.2196/63123 (PMC12707806; doi:10.2196/63123)
Supplement: Multimedia Appendix 4 [file humanfactors-v12-e63123-s004.pdf]

Multimedia Appendix 4: Comparison of baseline characteristics between the presence and absence of self-measurement

	Presence of self-measurement (n= 13)		Absence of self-measurement (n= 8)		*P*-value
	Mean	SD	Mean	SD	
Age, years	56.4	8.6	57.3	15.4	0.869
ME-BYO index, score	85.2	8.5	82.8	8.2	0.533
BMI, kg/m^2	23.7	4.6	23.9	4.3	0.925
Systolic blood pressure, mmHg	127.3	23.3	121.6	18.7	0.567
Diastolic blood pressure, mmHg	78.6	11.6	77.8	11.7	0.870
MPI, points	70.9	7.6	65.3	9.4	0.177
WHO-5, points	18.6	3.1	20.0	5.2	0.453
K6, points	7.5	2.2	6.3	0.5	0.119
Grip strength: right, kg	33.9	12.1	28.7	4.9	0.264
Grip strength: left, kg	32.9	12.2	25.8	6.0	0.144
Push-up, times/30 sec	14.7	5.7	17.6	7.5	0.349
5 times chair standing test, sec	6.7	1.2	6.1	1.5	0.317
Squat jump, cm	22.1	6.1	18.2	3.9	0.131
Countermovement jump, cm	24.8	6.6	20.9	3.8	0.155
Sit-and-reach test, cm	39.0	8.4	34.9	6.7	0.262
Muscle thickness, cm	3.2	0.5	3.5	0.5	0.287
Body fat, %	26.7	4.4	27.7	11.8	0.767
MVPA, min/day	74.1	37.9	88.8	45.0	0.432
Sedentary time, min/day	534.6	105.4	553.0	163.4	0.757

Unpaired t-test, SD: standard deviation, MPI: Memory Performance Index, MVPA: moderate-to-vigorous-intensity physical activity
